# School and community reopening during the COVID-19 pandemic: a mathematical modelling study

**DOI:** 10.1098/rsos.211883

**Published:** 2022-02-02

**Authors:** Pei Yuan, Elena Aruffo, Evgenia Gatov, Yi Tan, Qi Li, Nick Ogden, Sarah Collier, Bouchra Nasri, Iain Moyles, Huaiping Zhu

**Affiliations:** ^1^ Laboratory of Mathematical Parallel Systems (LAMPS), Department of Mathematics and Statistics, York University, Toronto, Canada; ^2^ Canadian Centre for Diseases Modeling (CCDM), York University, Toronto, Canada; ^3^ Department of Mathematics and Statistics, York University, Toronto, Canada; ^4^ Toronto Public Health, City of Toronto, Toronto, Ontario, Canada; ^5^ Department of Mathematics, Shanghai Normal University, Shanghai, People's Republic of China; ^6^ Public Health Risk Sciences Division, National Microbiology Laboratory, Public Health Agency of Canada, Quebec, Canada; ^7^ Department of Social and Preventive Medicine, Université de Montréal, Montréal, Quebec, Canada

**Keywords:** COVID-19, school reopening, community opening, transmission model, household structure, age structure

## Abstract

Operating schools safely during the COVID-19 pandemic requires a balance between health risks and the need for in-person learning. Using demographic and epidemiological data between 31 July and 23 November 2020 from Toronto, Canada, we developed a compartmental transmission model with age, household and setting structure to study the impact of schools reopening in September 2020. The model simulates transmission in the home, community and schools, accounting for differences in infectiousness between adults and children, and accounting for work-from-home and virtual learning. While we found a slight increase in infections among adults (2.2%) and children (4.5%) within the first eight weeks of school reopening, transmission in schools was not the key driver of the virus resurgence in autumn 2020. Rather, it was community spread that determined the outbreak trajectory, primarily due to increases in contact rates among adults in the community after school reopening. Analyses of cross-infection among households, communities and schools revealed that home transmission is crucial for epidemic progression and safely operating schools, while the degree of in-person attendance has a larger impact than other control measures in schools. This study suggests that safe school reopening requires the strict maintenance of public health measures in the community.

## 1. Introduction

Education has been severely disrupted by the COVID-19 pandemic. In most countries, the epidemic was controlled in spring 2020 by restrictive measures such as travel bans and closures of non-essential businesses and educational establishments. By mid-April 2020, 94% of learners worldwide were affected by the pandemic, representing 1.58 billion children and youth (C&Y), from pre-primary to higher education, in 200 countries [1]. Although school closures may help control the epidemic [2], they result in significant detrimental effects, including affecting children's learning and mental health, placing a high burden on the parents and reducing economic productivity [3]. Hence, policymakers worldwide have had to make difficult decisions about whether and how to reopen schools over the past several months. To date, there has been no easy answer or single standard [4].

Several studies have highlighted that to maintain control of the epidemic as we ease restrictive closures, stringent non-pharmaceutical interventions (NPIs) need to be in place. These include effective and rapid case detection with isolation of cases, effective and rapid contact tracing and quarantine of contacts, and maintenance of physical distancing and mask-wearing by the public [5–7]. Many countries reopened schools during late summer and early autumn 2020, adapting multiple control measures, such as cohorting approaches, distantiated single desks, as well as use of masks and hand sanitizer [8,9]. Risk mitigation measures in schools such as daily self-screening for symptoms and virtual attendance [10] were also enacted to help minimize infections in school-aged children and subsequent transmission of infections acquired in schools into the wider community. Still, the effectiveness of these and other mitigation strategies has yet to be examined.

The efficiency of school closures to mitigate infectious disease, including COVID-19, has been widely studied [11–18]. However, school closure is not a long-term solution, thus the safe reopening of schools is of critical importance to policymakers. Some studies have suggested that autumn 2020 was too soon to reopen schools [19], while others noted that with strict control of community transmission, schools have only a modest impact on virus spread [20–24]. Other measures, such as widespread testing, contact tracing and case isolation [3,25–27], as well as mitigation strategies including pre-semester screening [28] and household quarantine [27,29], may enable safe school reopening. For example, a recent study found that screening every 2 days using a rapid, inexpensive, and even poorly sensitive (greater than 70%) test, coupled with strict behavioural rules, was estimated to maintain a controllable number of COVID-19 infections and permit the safe return of students to campuses [18]. At the same time, it has been proposed that without strengthening NPIs, school reopening can contribute to secondary waves [3,27]. In addition, there is substantial evidence of secondary infections among household contacts [30–33]. In Toronto, household transmission is a key source of infection among C&Y, accounting for 20.5% of total infections, second only to community transmission [33]. While previous studies have highlighted the important role of school reopening in the trajectory of COVID-19, the transmission of infections acquired in schools into the wider community via transmission in households with children has not been examined. Furthermore, to date, there has been no literature on whether the resurgence of COVID-19 was driven by increases in contacts between C&Y and/or adults.

We developed a compartmental model to explore potential scenarios for the safe reopening of schools that incorporates age and household structure and different settings, coupled with varying levels of population-wide NPIs and school mitigation strategies. We used demographic and epidemiological data from Toronto, Canada, across multiple phases of escalation and de-escalation of contact-reducing policies for schools and community restrictions. This allowed us to define multiple infection transmission scenarios and carry out analyses simulating the outbreak trajectory if schools are reopened, or not, under different conditions. Our results can be adapted to any geographical region where over-time data on cases, demographics and policy phases are available.

## 2. Material and methods

### 2.1. Data sources

In order to allow results to be demonstrated as both daily and cumulative counts, during model development, we calibrated a deterministic transmission model using available surveillance data [34]. In fact, we used daily new confirmed cases by episode date, as well as cumulative confirmed cases and deaths, among adults and C&Y from 31 July to 23 November 2020, across multiple phases of escalation and de-escalation of public health policies including school closures and reopening (electronic supplementary material, table A1 and figure S1). We also used census data to inform the age and household structure of our model, with an average household size in Toronto of 2.4, and 38.39% of households with children [35]. In our simulations, the C&Y population accounts for 16.1% of the total population. Lastly, we used publicly available survey data to quantify the proportion of adults working from home and children learning virtually, as those behaviours may benefit the control of transmission [36]. Specifically, 68.34% of C&Y attended in-person learning [37], and 38.9% of adults worked from home [38].

### 2.2. Model description and assumptions

**Figure 1.**
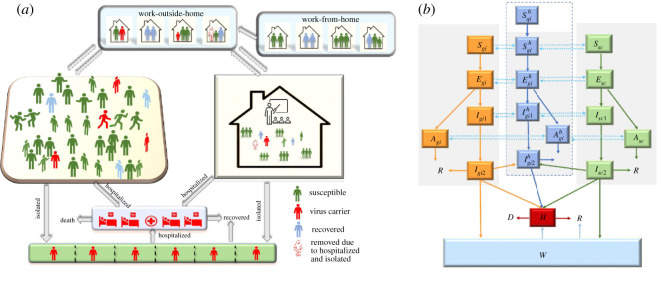
Transmission model with age and household structure. Panel (*a*) shows the activity and response of different structured population groups: household, schools and other community. Panel (*b*) shows a schematic diagram of the dynamics of COVID-19 in Toronto. Solid lines indicate movement between classes. Dashed lines represent the virus transmission routes. All individuals from work-outside-home (in subscript *g*), work-from-home (in subscript *q*), C&Y attending school in-person (*sc*) groups are in different disease states, Susceptible (*S_i_*), Exposed (*E_i_*), Infected (subclinical (*A_i_*), prodromal (*I_i_*_1_) or with symptoms (*I_i_*_2_)), Hospitalized (*H*), Isolated (*W*), Recovered (*R*) or Deceased (*D*). *i* indicates adults and C&Y population (*i* = *a,c*). The individuals in the home are labelled with superscript *h*, which is the sum of corresponding disease states in all the households. C&Y is children and youth.

We explored the impact of school opening using a deterministic age-household-location-structured Susceptible *S*- Exposed *E*- Asymptomatic *A*- (subclinical)—Infectious *I*_1_ (prodromal phase)—Infectious *I*_2_ (with symptoms)—Recovered *R*—Deceased *D* model framework, including individuals in self-isolation, who do not transmit the virus, through hospitalization (*H*) for the more severe cases, and fully isolated (*W*) for mild cases through testing and/or contact tracing (figure 1). Asymptomatic infections represent infections with no symptoms for the whole infection period. The population is classified into adults (20+) and C&Y (0–19), labelled as *a,c*, respectively. The model also incorporates additional complexity by including location structure (home, schools and community) and household structure (average household size, with/without children). There is evidence suggesting that work from home may reduce the risk of transmission [36,39]. Fisher *et al*. [36], for example, found that adults who received positive test results for COVID-19 were more likely to report going to an office or school setting, compared with those who tested negative [36]. Belzunegui-Eraso & Erro-Garcés [39] suggested teleworking as a measure to reduce COVID-19 transmission [39]. Hence, we further classify households as those where adults are working-from-home (WFH, in subscript *q*), with no social activity, or working-outside-of-home (WOH, in subscript *g*), with social activities in the community. The WFH households can convert to the WOH households following school reopening.

All the individuals belong to certain types of households with varied disease state composition. The household transmission is limited within household members and there is no household transmission if all the household members are not infected. The situation may change with the movement of individuals between the community, home and school. The individuals in WFH household all are assumed to be susceptible and isolated. The populations outside of household are composed of C&Y in the school and adults in the community both from WOH household, and the school transmission and community transmission are involved. The school transmission can occur when the school operated in the given schedule, while the household transmission and community transmission can occur every day. Population, community classifications and household compositions are reported in table 1. Demographic changes in the population (i.e. births, deaths) are ignored.

**Table 1.** Population, household and community classifications. Note: *n* is the household size.

	within household (in superscript *h*)				outside of household	
	WFH (in subscript *q*)		WOH (in subscript *g*)		school	community
	*n* = 2	*n* = 3	*n* = 2	*n* = 3	(in subscript *s*)	(in subscript *g*)
C&Y	0	1	0	1	C&Y attending in-person education	C&Y from WOH
adults	2	2	2	2	0	adults from WOH

Due to the influence of many factors such as people's personal characteristics, views on the epidemic, work types and household types, not all households will send C&Y back to school after the reopening, since in-person school attendance was optional. People need time to respond to the government policy, and they may gradually follow the policy. We assume that the process of C&Y returning to school and adults returning to work follows an exponential distribution [29] after schools opening on 8 September 2020, as we consider the returning behaviours of each household is a random event. Then we obtained the returning (back to school) rate of C&Y. The detailed definition of returning rate can be found in electronic supplementary material, §3.1. We modelled transmission over two periods: before and after schools reopened on 8 September 2020 [40]. Prior to school reopening, we assumed that all WFH individuals are susceptible, but, once C&Y go back to school, adults may return to work transitioning from WFH to WOH. While schools were closed, C&Y were at risk solely from infection in their household; however, after schools reopened, C&Y attending school in-person faced further risk of infection from within the school. If C&Y were also part of a WOH household, the risk of infection was possible from both family members and the community. Similarly, adults could have been infected by household members including children, or, if belonging to WOH, from the community as well. We additionally accounted for the impact of control measures specific to the school setting on transmission, including quarantine and contact tracing of school cases, mandatory self-screening procedures, and optional in-person attendance. The detailed model assumptions are presented in table 2. Also, the infectiousness among C&Y aged less than 19 years is assumed to be 50% of infectiousness in adults, based on available research suggesting lower susceptibility among children [3,41,42]. The contact matrix between adults and C&Y in the community is assumed to be symmetrical. The assumptions of the transmission risk in different locations are also defined in table 3. In addition, to mimic the transmission in the school, we included the school schedules into the model as there are no school transmissions during the weekend. Hence, our ODE model was solved within each day (split into 24 h).

**Table 2.** Model assumptions.

general	1	no birth, death or immigration
	2	the transmission can occur within the household and outside of the household (school and community)
	3	The population is divided into two groups: adults (marked by subscript *a*) and children and youth (C&Y, marked by subscript *c*). Households are further classified as those where adults are working-from-home (WFH marked by subscript *q*), or working-outside-home (WOH marked by subscript *g*). The community is made up of adults and *p_g_* proportion of C&Y from working-outside-home households. After school reopening, the schools setting contains C&Y (marked by subscript *sc*).
	4	each subpopulation is further divided into Susceptible (*S_i_*(*t*)), Exposed (*E_i_*(*t*)), Asymptomatic (subclinical) infection (*A_t_*(*t*)), Infectious pre-symptomatic (prodromal infection) (*I_i_*_1_(*t*)) and Infectious symptomatic (*I_i_*_2_(*t*))
	5	Both *A_i_*(*t*) and $I_{i1}(t)$ are infectious virus carriers and without symptoms. Individuals in *A_i_*(*t*) will never show symptoms, while individuals in $I_{i1}(t)$ develop into symptomatic classes ($I_{i2}(t)$) after a specified period of time.
	6	Symptomatic infections ($I_{i2}(t)$) are a source of infection until recovery. However, they may choose to self-isolate at home (or other places). If the quarantine is respected well enough, these infections will be fully isolated (*W*(*t*)).
	7	the fully isolated (*W*(*t*)), and the hospitalized (*H*(*t*)) who are severely affected do not contribute to infection transmission
household structure	8	all households contain n individuals and family members are homogeneously mixing i.e. contacting each other randomly
	9	the households are classified into two types: with one child (*n* = 3), without children (*n* = 2)
	10	the infection rate of the asymptomatic and symptomatic infectious individuals to the susceptible is the same among the household
	11	when there are no infections in a household, the family will no longer be involved in the transmission of COVID-19, unless the family members were infected in the community or school
school setting	12	all C&Y in the WOH household and part of the C&Y in the WFH household will come back to school after school reopening
	13	there are no symptomatic infections $I_{sc2}(t)$ every morning in the school due to the mandatory self-screening procedures, requiring symptomatic children to stay home
	14	the self-screen procedure might help detect infected C&Y who have no symptoms
	15	those infected C&Y with symptoms will be isolated
	16	the cohort with infected symptomatic C&Y will be quarantined at home
	17	schools are open between 9.00 and 15.00, Monday to Friday

**Table 3.** Transmission risk in different locations.

	transmission risk	children and youth	adults
household	home transmission risk	*β_q_*	*β_q_*
community	per contact transmission probability	*β_c_* = 0.5*β_a_*	*β_a_*
	contact rate	children and youth	adults
	children and youth	*c_cc_*	*c_ac_*
	adults	*c_ac_*	*c_aa_*
school	per contact transmission probability	*β_c_* = 0.5*β_a_*	
	contact rate	children and youth	
	children and youth	*c_sc_* = cohort size	

Household quarantine is one of the crucial control measures for mild cases. Given the importance of household transmission [27,29–32], we classified the households into different types based on the disease state of adults and C&Y in each household. The number of certain types of households (e.g. the household of two susceptible adults, one exposed C&Y) will change over time due to the dynamic of the epidemic in the household. We further calculated the variation in the number of different types of WOH households with different trajectories of disease progression, within-household transmission, transmission in the community and school, and the transition from WFH households to WOH households due to school reopening. The change rate of individuals in the WOH household can be calculated via the sum of the change rate of those individuals from all types of WOH households.

Upon consulting with public health stakeholders on our model assumptions, these two groups (adults, C&Y) were deemed to be most relevant for policymakers considering school and community reopening. Hence, in our model, there is no separation of adult populations into younger or older groups, and the school setting does not include adults, such as teachers and staff. The contacts between adults and C&Y in schools after school opened were considered through increases in the community (table 3). In addition, we distinguish households with and without C&Y in the degree of involvement in school transmission. The C&Y population in the model is informed by census data on the proportion of households with C&Y and the average household size in Toronto. As such, it may be underestimated due to the existence of the household of lone parents with one or more children or couples with two or more children. However, given that households with one person account for 32.34% of total Toronto households [35], this may overestimate the transmission risk in the household. Owing to these two possibilities, our method of modelling population composition should be appropriate and reasonable. It is important to note that our study only involved the wildtype that presented in autumn 2020 and does not distinguish between variants of the virus. New variants have been dominating case numbers since spring 2021 and this is discussed in our Scenario analysis. Details on variables, parameters, model structure and equations can be found in electronic supplementary material.

### 2.3. Time-varying intra-subpopulation risk

The time-varying intra-subpopulation risk (*R_t_*, type of effective reproduction number) was estimated in weekly sliding windows by using daily new cases of adults and C&Y [34], assuming a truncated normal distributed serial interval which was shortened over time by NPIs [43,44]. The mean of the serial interval ranged from 2.6 to 7.8 days with a standard deviation of 4.6–5.3 days [44]. In the simulations of hypothetical scenarios, we calculated the *R_t_* based on the daily new infections (including the asymptomatic and presymptomatic, and symptomatic infections).

### 2.4. Scenario analysis

We simulated the model from 31 July to 8 September following phase 1 of reopening and ran four scenarios. Scenario 1 represents the transmission risk prior to school reopening. Scenarios 2, 3 and 4 correspond to different NPIs being implemented after schools reopened. Scenario parameters are derived from the policy and transmission phases detailed in table A2 in electronic supplementary material. For example, scenario 2 uses the parameters of phase 2 (between 8 September and 2 October) and assumes that phase lasted the entire eight weeks. And phases differed in terms of both transmission levels and public health measures, including whether indoor and/or outdoor dining was permitted, the number of people allowed to gather, and retail capacity (for more detail, see electronic supplementary material, table A1). We compared the daily new cases of adults and C&Y population that our model predicts depending on whether schools were open or closed under different NPIs in the corresponding scenarios. We further considered the impact of increasing contacts in each of the adult and C&Y groups when school opens. Lastly, we investigated different thresholds for contact rates among adults for the safe opening of schools under different transmission probabilities and school reopening strategies.

### 2.5. Parameters and sensitivity analysis

Parameter values including the contact rates of adults and C&Y, household transmission risk in different phases, the efficiency of self-screening procedures, and the quarantine rate of symptomatic infection among adults and C&Y were estimated by minimizing the sum of squared differences between observed data and the model's estimates of daily and cumulative confirmed cases and deaths (electronic supplementary material, figure S2). All the parameters including those estimated from data and obtained from the literature are presented in electronic supplementary material, table A2.

We employed the Latin hypercube sampling/partial rank correlation coefficient (LHS/PRCC) method [45] to conduct sensitivity analysis of the model parameters on new cases in community and schools, to justify the effects of parameters uncertainty. We generated 2000 samples for the parameter that we are interested in, and the ones with a PRCC of magnitude greater than or equal to 0.5 were considered significant on the model output.

Analyses were carried out using Matlab (R2020a) [46] and R (v. 4.0.1) [47]. Data and code are available from https://github.com/yuanpei01/School_community_Covid.

## 3. Results

### 3.1. The risk of school reopening

**Figure 2.**
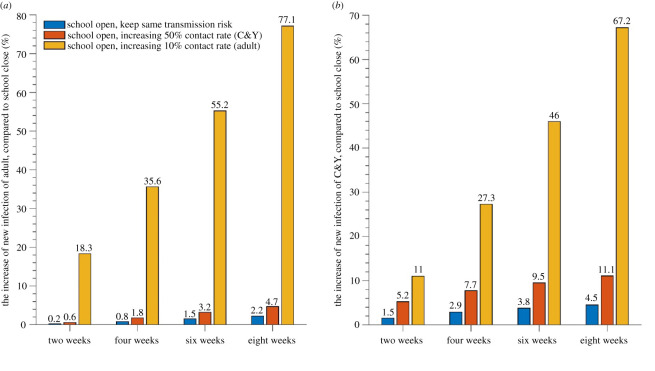
Percent increase in new cases among adults and C&Y under different scenarios, compared with when schools are closed. Per cent increase in new cases two, four, six and eight weeks after schools opened among adults (*a*) and C&Y (*b*) compared with a scenario with schools closed with no increases in contacts (blue bars), contacts among children increased by 50% (orange bars) and contacts among adults increased by 10% (yellow bars).

Our results show that opening schools increases case numbers in both adults and C&Y populations compared with schools remaining closed (figure 2; electronic supplementary material, table A3). Assuming that the contact rate of adults is the same before and after reopening, there is a larger percentage increase in the number of C&Y cases (4.5%) than adult cases (2.2%) eight weeks after school reopening. If contact rates are not the same pre- and post-reopening, figure 2 shows a significant change in cases compared with the scenario of schools remaining closed. If the contact rate among adults increases by 10% after schools reopen, the daily new cases in adults and C&Y increase significantly compared with schools remaining closed (increases of 77.1% and 67.2%, respectively, eight weeks after schools re-opened; figure 2*a,b* yellow bars). Given that opening schools is likely to increase contacts between adults, this may help explain the virus resurgence in Toronto in autumn 2020. If instead, the contact rates among C&Y had increased by 50%, there is much less of an impact on caseloads although an obvious increase in C&Y infections (figure 2*a,b*, orange bars).

### 3.2. School open versus closed and control strategy

**Figure 3.**
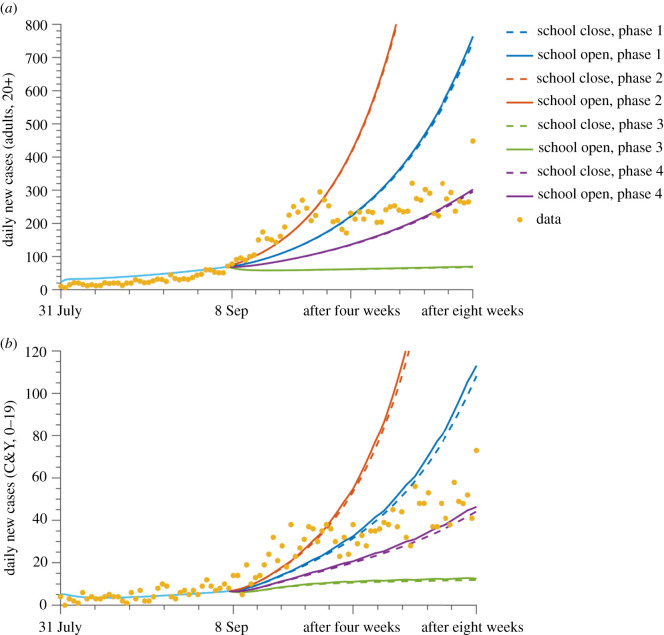
Scenarios where schools are open or closed after 8 September under different control measures. Daily new cases are reported within the adult (*a*) and C&Y (*b*) populations under different transmission levels, scenario 1 (blue), scenario 2 (orange), scenario 3 (green) and scenario 4 (purple) as described in the section on Scenario analysis. The solid and dashed line represents the scenarios of schools open or closed, respectively. Data are reported with yellow dots.

Using available data to define distinct scenarios of transmission and public health measures (see Material and methods and electronic supplementary material, table A1), our model can be used to simulate the epidemic trajectory if schools had been kept closed or reopened under different public health restrictions (figure 3; electronic supplementary material, table A3). Looking at the transmission risk levels across these scenarios, it is evident that the highest and sharpest increase of cases after 8 September 2020 occurs under scenario 2 conditions (orange lines), whereby indoor dining and gatherings are allowed, regardless of whether schools are open or closed, followed by scenario 1 (blue lines), scenario 4 (purple lines) and scenario 3 (green lines), where indoor gatherings are not allowed, and retail is restricted. However, regardless of community restriction levels, opening schools results in only a slight increase in cases among adults and C&Y, with a more pronounced increase in C&Y. Figure 3 shows that case numbers are relatively unchanged between opening and closing schools in each scenario. Thus, our model revealed that school reopening is not the key to outbreak resurgence, but rather it is the risk of transmission in the community that determines the trend of the epidemic. When community transmission is low, safe school opening is feasible (figure 3, green lines), under strict NPIs measures. By contrast, an exponentially increasing epidemic occurs irrespective of whether schools are closed or open with high community transmission risk due to weak NPIs (figure 3, orange lines).

### 3.3. Threshold of contact rates and safe reopening strategies

**Figure 4.**
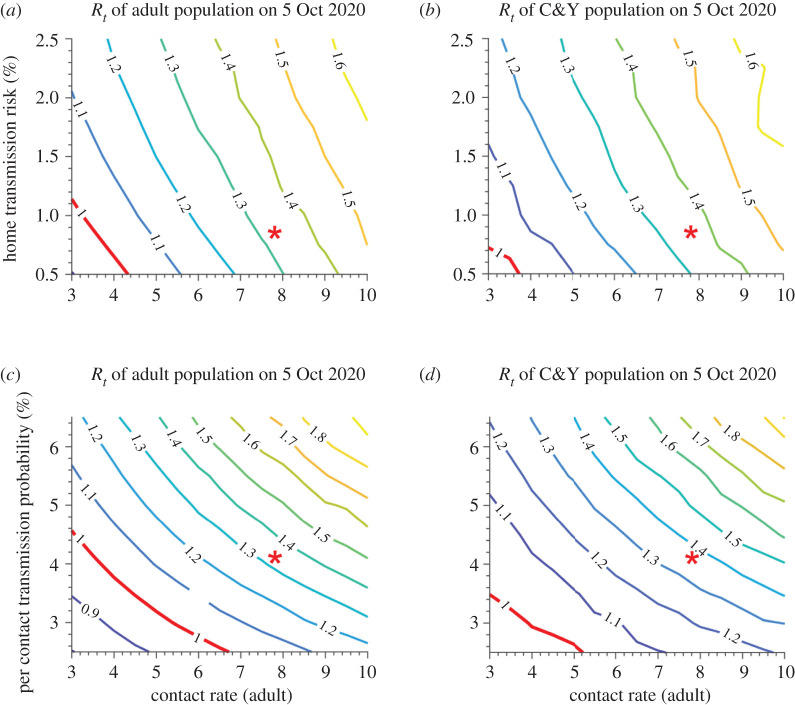
*R_t_* on 5 October 2020, with schools open, under varying household and community transmission risk. (*a,c*) Adult population; (*b,d*) children and youth (C&Y) population. Red star represents the level of phase 2; 5 October 2020 is the date of four weeks after school opening.

Overall, an increase in the home transmission risk and contacts will result in an increase in the *R_t_* in both age groups (figure 4). The *R_t_* of the C&Y population on 5 October 2020 (four weeks after school reopening) was slightly higher than that of the adult population, as they faced an additional risk of infection at school. In communities with higher home transmission probabilities (for example, those with a larger average household size), there is a higher risk of an epidemic rebound when schools are open. By contrast, with a per contact transmission probability of 4.1% (for the wildtype variant observed on 5 October 2020), in communities with a lower risk of infection at home (0.5%), controlling the contact rate of adults below three can keep the *R_t_* of adults and C&Y below one at four weeks following reopening (figure 4*a,b*).

Safe school reopening requires not only low contact rate, but also a reduction in the per-contact transmission probability. When the per-contact transmission probability increases to more than 4.8%, which could be a result of weaker adherence to self-protective behaviours or a result of the introduction new variants with more infectious transmissibility, it is difficult to prevent epidemic resurgence even with a low contact rate (less than three) among adults, regardless of schools being open or closed (figure 4*c,d*; electronic supplementary material, figure S3).

### 3.4. Sensitivity analysis

**Figure 5.**
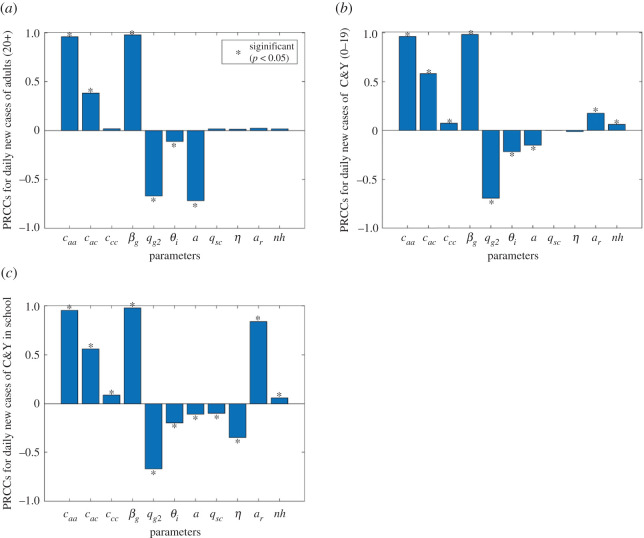
PRCC plots of transmission-related parameters on new cases among (*a*) adults, (*b*) C&Y and (*c*) C&Y in school. $c_{aa}$, $c_{ac}$ and $c_{cc}$ denotes the contact rate of adults–adults, adults–C&Y and C&Y–C&Y in the community. $\beta_{g}$ represents probability of transmission per contact among adults in the community. $q_{g2}$ is the quarantine rate of symptomatic infected adults. $\theta_{i}$ is the isolation rate of symptomatic adults' infection. *a*, proportion of infected with prodromal infection (1−*a*, the proportion of asymptomatic (subclinical) infection). $q_{sc}$ is the quarantine rate of symptomatic infected C&Y in the school. *η* is efficiency of self-screening procedures for detecting infected C&Y without symptoms. *a_r_* is the proportion of C&Y attending in-person education. *nh* is the cohort size in the school.

Sensitivity analysis (figure 5) on the contact rates shows that contacts among adults have a significant positive correlation on new cases, suggesting that an increase in these parameters will result in an increase in cases in both subpopulations, and in particular the C&Y population in school. Similar results for the probability of transmission among adults hold. The contacts among adults and C&Y are significant only for cases among the C&Y group. We also observed a significant negative correlation between the quarantine rate of symptomatic adults and case rates, implying that if more adults adhere to quarantine, the infection will spread less, confirming the crucial role played by the contacts among adults shown in figure 2. In addition, the proportion of asymptomatic infections is significantly positive associated with the adults' infections, while showing less importance on the C&Y infections.

Furthermore, the quarantine rate of symptomatic infected C&Y in the school and the efficiency of self-screening procedures are negatively correlated with the number of infections in the school, while the rate of in-person attendance and cohort size in schools are positively associated with cases in the school. And in-person attendance appears to be a crucial driver for cases in schools.

## 4. Discussion

Our novel method of modelling COVID-19 transmission dynamics takes into account age structure and household transmission, allowing us to examine risks within and between households, communities and schools, and to explore whether the school reopening was responsible for the autumn 2020 virus resurgence in Toronto. We found that school opening can be safe under strict NPIs (for example, restrictions on indoor dining, retail and gatherings), though a slight increase in infections among adults and C&Y within eight weeks following school reopening is to be expected (2.2% and 4.5% increase, respectively). Whether the reopening of schools can result in virus resurgence depends largely on countermeasures in the community. Our model predicts that the increase in case counts that occurred when schools opened were primarily due to the rise in contacts between adults returning to work and social activities after schools opened. Importantly, it was not due to an increase in contacts between C&Y. Sensitivity analyses further confirmed that adults represent the age group most affecting the spread of the infection, supporting our finding of rapid increases in case counts when the contact among adults is increased even slightly. Overall, reducing the contact rate in the community is more effective in mitigating the epidemic than school closures in terms of cases in adults, while household transmission plays a larger role for C&Y cases, especially in communities with a larger average household size.

The safe operations of schools during the pandemic necessitates risk mitigation measures. We examined the effectiveness of those measures in a sensitivity analysis and found that in-person versus virtual attendance was the biggest driver of infection in school; with high in-person attendance, additional measures are needed to mitigate the outbreak. Strict public health measures (restricting gatherings, maintaining social distancing, etc.) to reduce contact rates, and self-protection behaviours (the use of masks and disinfectant, hand washing, etc.) to reduce the probability of transmission per contact, should be in place at the same time. Beyond stringent NPIs in the community, public health communication should also emphasize preventative practices, such as hand-washing, for students returning from school. Furthermore, widespread testing, tracing and isolation of mild cases have been shown to be effective in decreasing virus spread [3,20,25–27,32]. Therefore, we conclude that overall, while the size and transmission in households dictates the allowable contacts to prevent community outbreaks, the key to allowing safe school opening is the maintenance of strict NPIs to reduce community spread.

Worries of school opening during the pandemic stem from the potential increased transmission risk due to school-related activities and other social activities. The parents are concerned about the infections in the C&Y group, and it may also bring the risk into the household, hence exacerbating the community transmission risk. Therefore, the higher community transmission may be itself due to the reopening of school. However, the labour force in educational service only accounts for 7.1% of the total labour force in Ontario [48]. The school-related activities cannot be entirely blamed for the whole increased community transmission risk after school reopening. The rise of contact in adults returning to work and the increase of social and leisure time are also the major responsible drivers, verified by our model. Moreover, the rise in contacts in adults and C&Y is inescapable after schools reopened, which further illustrates the importance of the strict NPIs to allow safe reopening, although the slight increase of cases in C&Y. In the resurgence in Europe, it was also found that the smaller effect of closing schools and the stringent safety measures made schools safer compared with the first wave [49].

The C&Y are facing a higher risk after school reopening in autumn 2021 under the circumstance of the higher transmissible Delta variant and relaxation of NPIs, and it is more challenging to maintain safe school operation during the pandemic. Although the vaccine coverage in the adult group is high, the transmission is worrying given the low efficacy of the vaccine against the Delta variant and the waning immunity [50,51]. On the other hand, the Center for Disease Control and Prevention of the United States promotes vaccination for children 5 through 11 years old [52], which may further reduce the transmission risk among C&Y in the schools. Nevertheless, with the limited reduction of transmission risk due to the vaccine [50,53], rigorous NPIs are still essential to allow safe reopening. Future modelling that incorporates variants of concern and vaccination rollout will allow for a deeper analysis of safe school opening during the transition of pandemic to endemic.

Our analyses have some limitations. First of all, the incorporation of household structure has significantly increased the complexity of our model, thereby limiting our ability to include households of larger household sizes. The composition of disease states in the household substantially increases in complexity with increasing household size. Our current model with household sizes 2 and 3 has more than 300 equations. Incorporating additional sizes will dramatically increase the number of equations and will result in technical difficulties in running the model. Our model description can allow other researchers to apply our methods to their own contexts, however. In addition, the intra-subpopulation risks were investigated by calculating its effective reproduction number based on the daily new infections of subpopulation and it may be overestimated because the mixed infection sources are not considered in the statistical model. However, it is a great indicator to inform the real-time spreading in each population along with the policy changes. Moreover, the indirect effects of school reopening, for example, more healthcare workers returning to work and thereby reducing mortality [54–56], have not been explicitly modelled in our study. Future research is needed to explore this and other trickle-down effects of school opening.

## 5. Conclusion

In summary, our findings suggest that the combination of stringent public health measures to control transmission in the community, mitigation efforts in schools, and efforts to minimize transmission in the household can allow for the safe reopening of schools. However, with schools open, the increasing contacts among adults in the community can contribute to a large-scale surge in the epidemic in the absence of adequate NPIs that would prevent gatherings and contacts in indoor spaces. With both settings open, stringent preventative practices for students returning home from school and adults returning from work to reduce cross-transmission between the household, the community and the school are warranted.

## Data accessibility

The data used for this study are provided by Toronto Public Health and are publicly available at the following link: https://open.toronto.ca/dataset/covid-19-cases-in-toronto/ (accessed 4 December 2020). Also, data and code are available from https://github.com/yuanpei01/School_community_Covid. The data are provided in electronic supplementary material [57].
